# *Eleutherodactylus* frogs show frequency but no temporal partitioning: implications for the acoustic niche hypothesis

**DOI:** 10.7717/peerj.496

**Published:** 2014-07-22

**Authors:** Luis J. Villanueva-Rivera

**Affiliations:** Forestry and Natural Resources, Purdue University, West Lafayette, IN, USA

**Keywords:** Acoustic niche hypothesis, *Eleutherodactylus*, Community, Puerto Rico, Bioacoustics

## Abstract

Individuals in acoustic communities compete for the use of the sound resource for communication, a problem that can be studied as niche competition. The acoustic niche hypothesis presents a way to study the partitioning of the resource, but the studies have to take into account the three dimensions of this niche: time, acoustic frequency, and space. I used an Automated Digital Recording System to determine the partitioning of time and acoustic frequency of eight frogs of the genus *Eleutherodactylus* from Puerto Rico. The calling activity was measured using a calling index. The community exhibited no temporal partitioning since most species called at the same time, between sunset and midnight. The species partitioned the acoustic frequency of their signals, which, in addition to the microhabitat partitioning, can provide some insight into how these species deal with the problem. This data also suggest that monitoring projects with this group should take place only before midnight to avoid false negatives.

## Introduction

The problem of how species in acoustic communities deal with the limited bandwidth of the acoustic resource can be studied as niche competition. In this case the resource is a single medium of communication: air for terrestrial communities and water for aquatic and marine systems. In particular, there are three dimensions in which communities can partition this niche: in time, acoustic frequency and space (Garcia-Rutledge & Narins, 2001; Wells, 2007).

Partitioning of the acoustic frequency and timing of the signals has been subject of study in anuran communities for decades (Littlejohn, 1965; Chek, Bogart & Lougheed, 2003; Steelman & Dorcas, 2010). This partitioning, formally posited as the acoustic niche hypothesis, may help explain community structure when each community assembles itself in ways to reduce competition for the sound (Krause, 1993; Farina et al., 2011).

Most studies on anuran acoustic communities have been limited to a single dimension, making generalizations very hard to reach (Wells, 2007). An assumption that is often made is that the whole community is stable, where the partitioning is caused by competition and displacement of the acoustic frequency of their calls (Wells, 2007). However, without data on the temporal partitioning it is not possible to determine that the competition pressures are enough to drive a change. This is particularly difficult in assemblages at a same study site that are highly variable (Guyer & Donnelly, 2005; Wells, 2007).

The acoustic community of the Puerto Rican *Eleutherodactylus* frog species was described as having both temporal and acoustic partitioning (Drewry, 1970; Drewry & Rand, 1983). However, these patterns were generated systematically for five species and subjectively, from field notes, for the other nine species. It is not clear if these patterns are accurate enough to determine the peak of activity for each species and they did not seem to match observations in the field (LJ Villanueva-Rivera, pers. obs., 2002).

The objective of this study was to test the acoustic niche hypothesis in anurans by determining if there is temporal and acoustic frequency partitioning in the calling activity of highland species of *Eleutherodactylus* frogs from Puerto Rico. These patterns were determined using Automated Digital Recording System (ADRS (Acevedo & Villanueva-Rivera, 2006)). This community exhibits microhabitat partitioning but this dimension was not included in this study (Stewart & Woolbright, 1996).

## Methods

**Figure 1:**
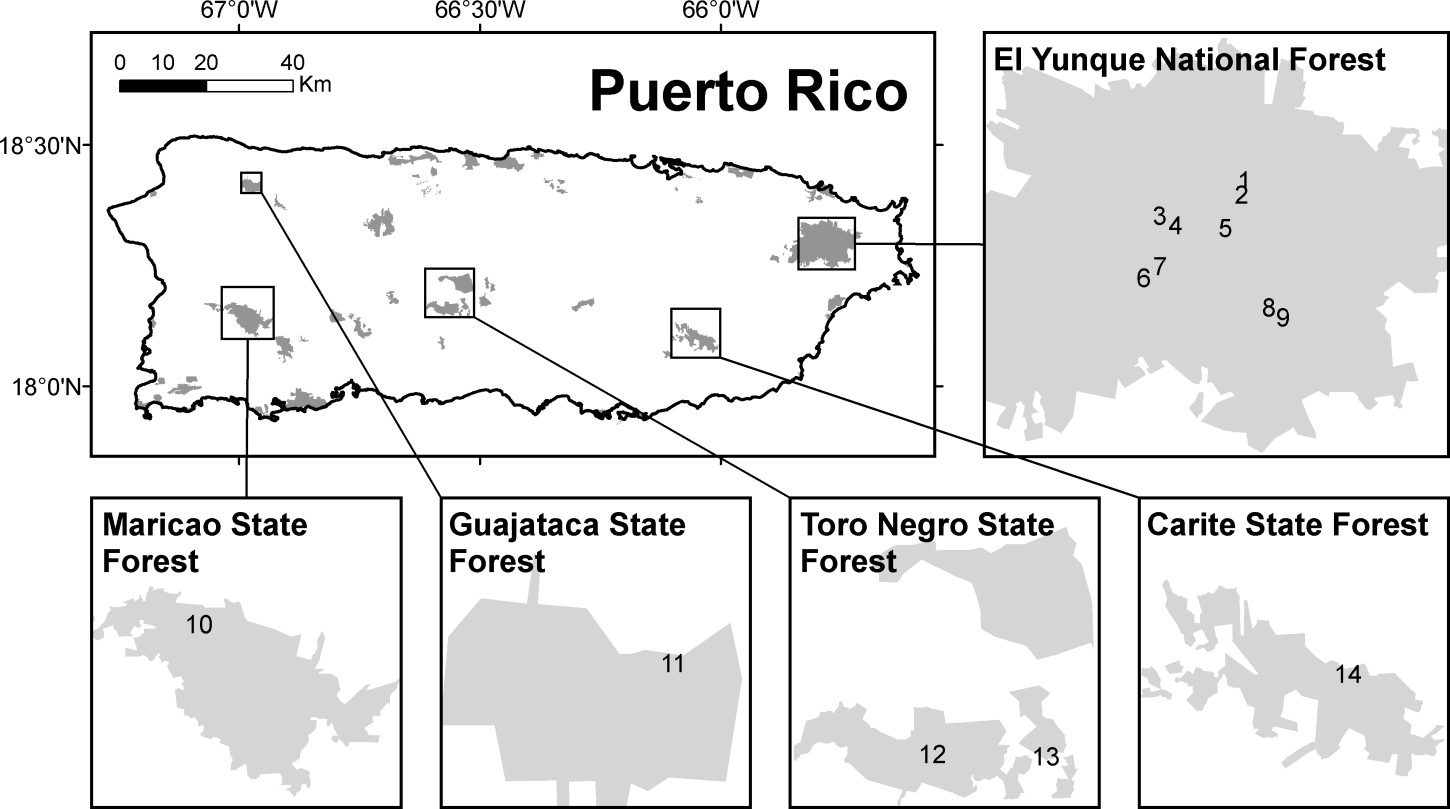
Locations surveyed acoustically for frogs in Puerto Rico. Shaded areas represent protected areas of the island. The numbers represent the locations as listed in Table 1.

Populations of highland Puerto Rican *Eleutherodactylus* frogs were sampled at 14 sites with ADRS to determine the calling activity for each species between 2003 and 2004 (Fig. 1). At each site, the sensor was deployed until the batteries were depleted, which usually lasted 5 days unless there was some equipment failure. With the exception of the Carite State Forest site, all sites were surveyed once (Table 1).

**Table 1:** Sites, dates sampled, and species detected but not used for the analysis in this study.

Site no.	Site^*^	Dates	Species not analyzed
1	EYNF Road 191, km 9.3	30/Oct/03–1/Nov/03	*Eleutherodactylus antillensis*
			*E. wightmanae*
2	EYNF Road 191, km 9.1	28/Apr/04–3/May/04	*E. wightmanae*
3	EYNF Mt. Britton Spur	28/Apr/04–3/May/04	
4	EYNF Mt. Britton Tower	30/Oct/03–3/Nov/03	*E. unicolor*
5	EYNF Palo Colorado	11/Apr/04–16/Apr/04	
6	EYNF Tradewinds Trail	15/Jul/04–20/Jul/04	*E. locustus*
			*E. wightmanae*
			*Leptodactylus albilabris*
7	EYNF Tradewinds Trail	11/Apr/04–16/Apr/04	*E. locustus*
8	EYNF Pico del Este	28/Apr/04–3/May/04	*E. gryllus*
			*E. locustus*
			*L. albilabris*
9	EYNF Pico del Este	12/Aug/04–17/Aug/04	*E. unicolor*
			*L. albilabris*
10	Maricao State Forest	7/Feb/04–11/Feb/04	*E. richmondi*
11	Guajataca State Forest	19/Apr/04–24/Apr/04	*E. antillensis*
12	TNSF	16/Jun/04–21/Jun/04	
13	TNSF-Lago Guineo	19/Feb/04–24/Feb/04	*E. portoricensis*
			*E. wightmanae*
			*L. albilabris*
14	Carite State Forest	19/Mar/04 – 21/Mar/04	
		30/Mar/04–4/Apr/04	

**Notes.**

*
EYNF El Yunque National Forest TNSF Toro Negro State Forest

The ADRS consisted of a Nomad Jukebox 3 digital player and recorder (Model DAP-HD0003, Creative Labs, Inc, California), a portable preamplifier (Model SP-PREAMP, The Sound Professionals, Inc., New Jersey) and an electret condenser microphone (Model ECM-MS908C, Sony Electronics Inc, California). The microphone was placed at approximately one meter from the ground. The system was controlled with a microcontroller (model MSP430-P-1121M, Olimex Ltd., Plovdiv, Bulgaria), which triggered commands to the recorder to record 1 min every 30 min. Each sample was stored as a .wav file using a sampling rate of 48 kHz.

Each recording was listened to with headphones to determine the species calling and their respective activity level. The recordings were loaded to the AUDITION software (ver. 1.0; Adobe Systems, Inc., California, USA) to visualize the species’ calls in the spectrogram that were not audible due to interference by other loud species, usually by *Eleutherodactylus coqui* (Villanueva-Rivera, 2007). Each species has a distinct call which has been described and published (Rivero, 1998). After listening to a recording once, if the spectrogram indicated the possibility of another species that was not heard, the audio was filtered to remove the range of frequencies of other species and listened to again.

The activity level of each species in each recording was categorized using the Amphibian Calling Index (ACI). The ACI can have four values: (0) represents no individuals calling; (1) a few individuals calling with no overlap between the calls; (2) there is some overlap; and (3) a full chorus (Nelson & Graves, 2004). Each 1-min recording had an ACI value for each species. This resulted in 25 ACI values for each species for each night, one every 30 min between 1800 and 0600 h.

To determine temporal partitioning, the ACI values for each species were analyzed using a Kruskal-Wallis test with the null hypothesis that the calling activity was uniform during the night, between 1800 and 0600 h (Sokal & Rohlf, 1995). The noise from rain or wind in several recordings made it difficult to determine the ACI for the species. These recordings were not included in the analysis, which resulted in dissimilar sample size between the 25 periods during the night. In some sites, the average ACI of some species was lower than 1. When this was the case, the data for the species at the site was not included in the analysis.

To determine acoustic partitioning of the populations, I used the recordings made at the time with the peak of calling activity for most species, at 2000 h. The peak in their activity was used to compare the full chorus instead of a subset of the chorus that would be calling at non-peak times. In addition, the same time was used to avoid the variability due to temperature changes during the night.

The frequency range of the *Eleutherodactylus* species present in these recordings was measured using the software Pumilio (Villanueva-Rivera & Pijanowski, 2012). When species had an overlap in frequency, their calls were compared for each pair to measure the proportion of overlap in the frequency range. Because frequency varies by temperature and elevation (Narins & Meenderink, 2014), and therefore by site, each pair-wise comparison was made by site and then aggregated as percent of overlap across sites.

Statistical analyses were performed using R (v. 2.5.1, R Development Core Team, Vienna, Austria) and *α* = 0.05. All the recordings and data tables are stored in Figshare (doi: 10.6084/m9.figshare.806302).

This research was conducted in the state forests under the authorization of the Department of Natural and Environmental Resources of Puerto Rico (02-IC-068) and at the El Yunque National Forest under the authorization of the United States Forest Service (CNF-2038). Since there was no collection or manipulation of individuals, approval by the Institutional Animal Care and Use Committee was not required.

## Results

I detected 10 species of frogs in 1,550 audio recordings at the 14 sites surveyed (Table 1). Two species, *Leptodactylus albilabris* and *Eleutherodactylus antillensis*, with a widespread distribution on the island (Rivero, 1998), were heard occasionally at some sites and were not included in the analysis. One species, *Eleutherodactylus coqui*, a generalist species, was present at all sites.

### Temporal partitioning

**Figure 2:**
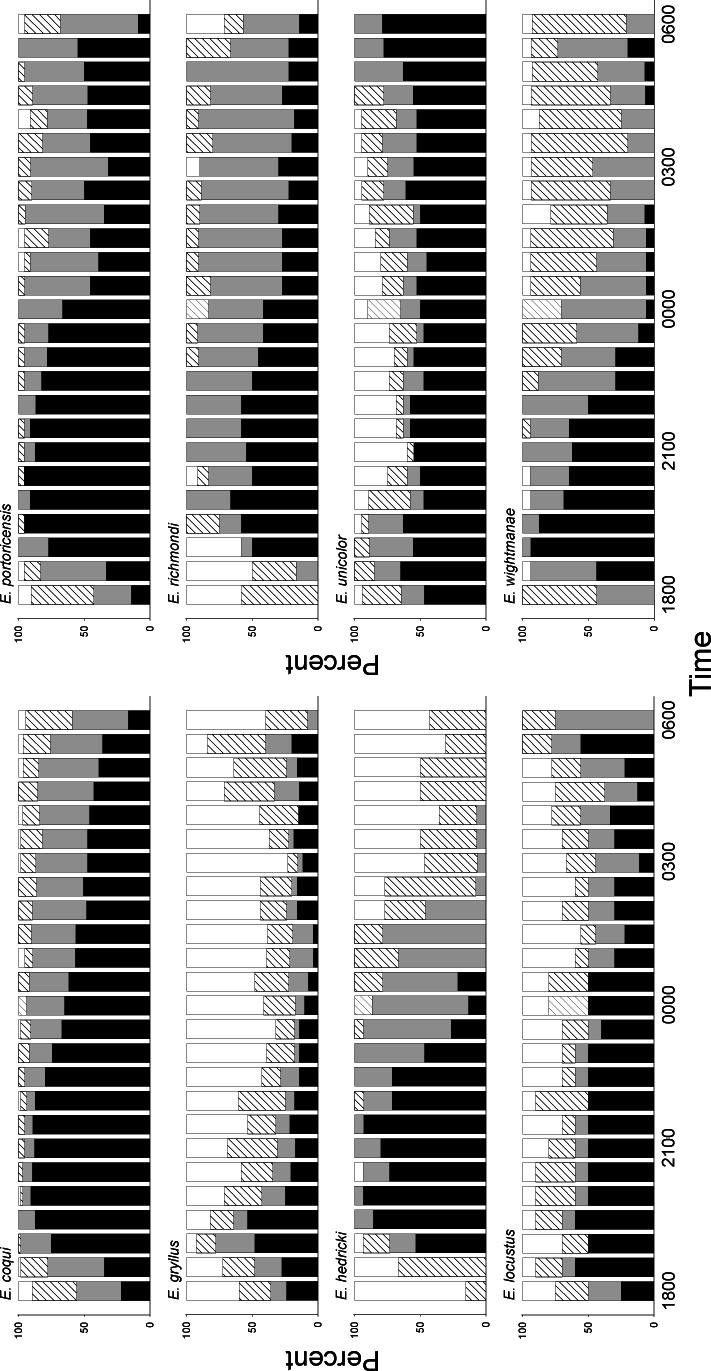
Percentage of calling activity level measured as Amphibian Calling Index (ACI) for eight species of *Eleutherodactylus* frogs from Puerto Rico. White bars represent ACI, 0 (no individuals calling); diagonal lines represent ACI, 1 (a few individuals calling with no overlap); gray bars represent ACI, 2 (there is some overlap); and black bars represent the percentage of samples with ACI, 3 (full chorus).

The pattern of calling activity was estimated using the Amphibian Calling Index (ACI) (Nelson & Graves, 2004). The pattern during the night for six of the eight species was significantly different at some part of the night (Table 2). Five species had their peak of activity between sunset and midnight: *E. coqui*, *E. hedricki*, *E. portoricensis*, *E. richmondi*, and *E. wightmanae*, measured as a higher proportion of samples with ACI values of 2 or 3 (Fig. 2). The calling activity of these five species was highest between 1900 and 2100 h and declined steadily after midnight. In the case of *E. gryllus*, the species had a short peak of activity between sunset to about 2000 h. Two species, *E. locustus* and *E. unicolor*, showed no significant difference in their activity during the night.

**Table 2:** Results of the Kruskal-Wallis test on the uniformity of the calling activity during the night for each *Eleutherodactylus* species in this study. Some samples had noise from rain or wind and were not included, which resulted in different sample sizes for some time periods.

Species	H value	Range of *n*
*Eleutherodactylus coqui*	319.4^*^	54–67
*E. gryllus*	94.7^*^	21–29
*E. hedricki*	268.9^*^	12–15
*E. locustus*	15.3	8–10
*E. portoricensis*	154.8^*^	19–24
*E. richmondi*	77.6^*^	7– 12
*E. unicolor*	26.4	17–20
*E. wightmanae*	190.6^*^	14–17

**Notes.**

* *p* < 0.001.

Three species had a small peak of activity during the last hours of the night (Fig. 2). The species *E. gryllus*, *E. portoricensis*, and *E. wightmanae* increased their activity two hours before sunrise from the declined activity level of the hours after midnight. These peaks were smaller than the main peak before midnight.

To illustrate some of these patterns, an example series of audio files recorded every hour, between sunset and sunrise, is available as Dataset S1.

### Acoustic frequency partitioning

**Figure 3:**
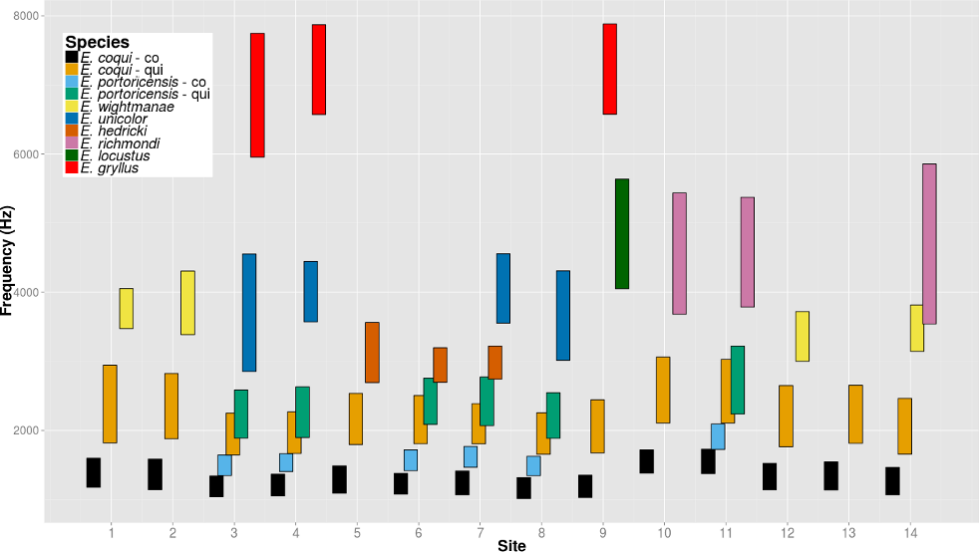
Frequency range occupied by each species at each site. Both *E. coqui* and *E. portoricensis* have two notes, known as “co” and “qui”, that were measured separately.

From the whole dataset, the 64 recordings made at 2000 h were analyzed to determine acoustic partitioning. In all sites with more than one species, most species exhibited frequency partitioning (Fig. 3), where the frequency range of their signals did not overlap. Between *E. coqui* and *E. portoricensis* there was a large overlap in one of the notes. Their first note, “co”, did not overlap. Their second note, “qui”, showed an overlap that averaged 63.9% (54.5–85.9%) of the frequency range of the note of *E. coqui* and 57.8% (45.2–80.3%) of the frequency range of the note of *E. portoricensis* (*n* = 6).

Two other cases showed some overlap to a lesser degree. The calls of between *E. wightmanae* and *E. richmondi* at the Carite State Forest (Site 14 in Table 1) overlapped 42.0% (10.3–83.0%) and 11.5% (4.2–21.0%) respectively (*n* = 5). At the El Yunque National Forest, the call of *E. hedricki* and the “qui” note of *E. portoricensis* showed some overlap at the Tradewinds Trail sites (Sites 6 and 7 in Table 1). The overlap in the call of *E. hedricki* was 9.7% (0–17.9%) and for the “qui” note of *E. portoricensis* was 7.0% (0–10.9%).

## Discussion

### Temporal partitioning

The results from this study indicated that the level of activity of six of eight highland *Eleutherodactylus* species studied was not uniform during the night. Most species called from sunset to midnight, with a peak around 2000 h. These results suggest that this anuran community does not have temporal partitioning of their calling activity during the night. Seasonal differences will need to be studied using long-term datasets.

A previous study found some temporal partitioning in several species at El Yunque National Forest (Drewry & Rand, 1983). In particular, two species in that study, *E. portoricensis* and *E. richmondi*, were calling later that the other species in the night. However, the data used in that study were collected using different methods and from field notes, not from a systematic survey during the night. Another study using ADRS in a palustrine herbaceous wetland in Puerto Rico also found temporal clustering in four *Eleutherodactylus*, among them the generalist *E. coqui*. The four species at this site exhibited a peak of calling activity also at 2000 h (Ríos-López & Villanueva-Rivera, 2013), which is the first period of complete darkness. The sunset in Puerto Rico happens between 1800 and 1900 h (Ríos-López & Villanueva-Rivera, 2013).

It was expected that species should limit their calling activity to a period when its benefits (attracting females) are outweighed by its costs (energy expenditure, reduced foraging, and predation risk). Reproductive success of *E. coqui* is determined only by calling effort (Townsend & Stewart, 1994). In several studies that compare the energetic cost of calling, the metabolic rate increases up to ten times, making it a very energetically expensive activity (Gerhardt, 1994; Wells, 2001). In *E. coqui*, males reduce the number of prey they consume while calling (Woolbright & Stewart, 1987) and the energy requirements of calling stop their growth (Woolbright, 1989). Predation on *Eleutherodactylus* by owls (*Megascops nudipes*) has been reported (Zelick & Narins, 1982), so it is possible that these predators may sometimes use the call of the males to hunt them.

Acoustic surveys are a standard method for anurans (Zimmerman, 1994; Rödel & Ernst, 2004; Dorcas et al., 2009), but communities with temporal clustering present some problems. Very loud species, like *E. coqui*, can mask other species present at the sites (Villanueva-Rivera, 2007). Audio recordings can be a better method for monitoring these species, in particular when combined with automated identification (Aide et al., 2013). Results from this study suggest that acoustical monitoring of Puerto Rican *Eleutherodactylus* species should take place from sunset to midnight, when most of the species are highly active, with recorders to reduce false negatives due to masking. Furthermore, surveys conducted after midnight should be avoided as low calling activity levels could be due to the time and not a local extinction or a declining population. Special attention should be given to cases like *E. gryllus*, with a very short peak of calling activity limited to the first two hours of the night.

### Acoustic frequency partitioning

The acoustic community of *Eleutherodactylus* species exhibited partitioning in the acoustic frequency of their sound signals. The calls of *E. wightmanae* and *E. richmondi* showed some overlap, however the calls are very different, which could reduce the pressure for frequency displacement. The call of *E. wightmanae* is a repetition series of a note while the call of *E. richmondi* is a short click with a very broad range in frequency (Rivero, 1998). In the other case of overlap, the species have spatial partitioning. *Eleutherodactylus portoricensis* is found in the understory up to 3 m of the ground, while *E. hedricki* only calls from holes in old branches near the canopy (Stewart & Woolbright, 1996).

The large overlap in the second note, “qui”, of the call of *Eleutherodactylus coqui* and *E. portoricensis* found in this study deserves further study, in particular because the species do not show temporal or spatial partitioning. Both species partition the frequency space of their “co” note. In invasive *E. coqui* populations in Hawaii, the “co” has very little inter-individual variation, while the “qui” note seems to be more variable (Benevides & Mautz, 2013). This high variability and overlap between sympatric *E. coqui* and *E. portoricensis* could indicate that this is not an important signal for distance communication since evolution has not separated this signal as the others. The two-note call of *E. coqui* has been studied in some detail. The first note, “co”, seems to be important to maintain distance between calling males and to establish their territory, while the second one, “qui”, is used to attract females (Narins & Capranica, 1976; Narins & Capranica, 1978; Zelick & Narins, 1982).

A study that tested the effect of two levels of density of sympatric *E. portoricensis* on the dominant frequency of the notes of *E. coqui* found that the “co” did not change, while the acoustic frequency of the “qui” was lower in sites with high densities of *E. portoricensis* (Luther et al., 2012). *E. coqui* might be trying to avoid masking of the noise or the higher densities of *E. portoricensis* could be triggering a suppression of the call in individuals with higher overlap in the frequency range (Zelick & Narins, 1982). The mechanism that is driving this effect of lower frequency could be studied by determining whether the females select males with lower frequency due to masking or if males that call at higher frequencies have less reproductive success because they are suppressed by the heterospecific call. In *Hyla cinerea*, the presence of *H. gratiosa* was related to displacement in female preference and in the advertisement call of the males (Höbel & Gerhardt, 2003). However, a study with sympatric populations of two *Pseudacris* species showed that the character displacement was variable among sites (Lemmon, 2009).

Several studies seem to indicate that acoustic frequency partitioning in anuran communities is not common. In a review of 11 assemblages, only 3 showed acoustic partitioning (Chek, Bogart & Lougheed, 2003). However, there was no data on temporal or spatial partitioning in most assemblages and some included data from a large geographical region. Other factors can make it harder to study, including separation of the acoustic frequency due to factors other than competition for the acoustic resource (Gerhardt & Schwartz, 1995).

A null model of a 7-species community in a pond in Costa Rica did not find differences in the frequency partitioning with random assemblages (Guyer & Donnelly, 2005). However, this study documented 28 combinations of up to 6 species during 47 sampling nights. This large variability of species may not be enough selective pressure to induce displacement of the acoustic frequencies (Pfennig & Pfennig, 2009).

In a community of five anurans in the Andes of Colombia, three of them *Eleutherodactylus*, the four species that were nocturnal had their peak of calling activity between one and two hours after sunset (Lüddecke et al., 2000). The species partitioned both the calling sites used and the acoustic frequency range (Lüddecke et al., 2000). In a Thailand assemblage of 11 species in 3 families, a study found partitioning in acoustic frequency, timing and space (Garcia-Rutledge & Narins, 2001).

In a community of 13 species in Brazil studied in permanent and temporary ponds and swamps, species that did not partition in space, partitioned their acoustic signals (Santos & Rossa-Feres, 2007). In turn, species that had similar calls partitioned their use of space (Santos & Rossa-Feres, 2007).

## Conclusions

This study provided support for the acoustic niche hypothesis in anurans. However, it seems the partitioning of the acoustic resource of anurans is not a simple phenomenon to study and previous studies have been too limited to provide evidence for or against it. Although there have been suggestions for the study of this problem using null models and by comparing sympatric and allopatric communities (Gerhardt, 1994), few studies have used this type of comparison and the ones that have do not present a clear consistent result in anuran acoustic communities (Chek, Bogart & Lougheed, 2003; Lemmon, 2009; Luther et al., 2012). The models against which the data would be tested need to take into account the special cases of species will multiple notes. Some notes may be selected for partitioning, while others not, like in the case of the “qui” notes of *E. coqui* and *E. portoricensis*.

Confounding factors, like evolutionary history, in particular when dealing with several families of anurans that congregate at the same site, and diversity of reproductive strategies should be taken into account in future studies to determine which are the determining factors in these acoustic community assemblages. Conservation efforts should also take these sources of competition into consideration when selecting sites for re-introduction.

The acoustic niche hypothesis will need to be studied in all its dimensions, time, acoustic frequency and space. In this study the species exhibited no partitioning in the time dimension but partitioning in the acoustic frequency dimension. The study of the spatial dimension will depend on what is known of the behavior of the species. For either of the other two dimensions, acoustic frequency (Chek, Bogart & Lougheed, 2003) or time (Steelman & Dorcas, 2010), may not provide enough data, or the conclusions might not be generalizable due to the unknown influence of the other dimensions.

This acoustic community of *Eleutherodactylus* frogs from Puerto Rico present a good opportunity to study the acoustic niche hypothesis and the evolution of call displacement. All the species of Puerto Rico are closely related (Heinicke, Duellman & Hedges, 2007; Hedges, Duellman & Heinicke, 2008), most call at the same period of the night, and they are the majority of the anuran fauna in the island. These qualities reduce the added complexity of previous studies that compared communities comprised of several families (Gerhardt & Schwartz, 1995; Chek, Bogart & Lougheed, 2003).

## Supplemental Information

10.7717/peerj.496/supp-1 Dataset S1 Example series of hourly audio recordings Open the file index.html to see a series of recordings made every hour between 13 Aug 2004 18:00 to 14 Aug 2004 06:00
Each file is presented as a wave file, with an audio player in index.html, and the spectrogram of the file.

## Additional Information and Declarations

### Field Study Permissions

The following information was supplied relating to field study approvals (i.e., approving body and any reference numbers): This research was conducted in the state forests under the authorization of the Department of Natural and Environmental Resources of Puerto Rico (02-IC-068) and at the El Yunque National Forest under the authorization of the United States Forest Service (CNF-2038).

### Data Deposition

The following information was supplied regarding the deposition of related data: Figshare: 10.6084/m9.figshare.806302.
