# Supplementary material for: Eleutherodactylus frogs show frequency but no temporal partitioning: implications for the acoustic niche hypothesis
Source: PeerJ. 2014 Jul 22;2:e496. doi: 10.7717/peerj.496 (PMC4121589; doi:10.7717/peerj.496)
Supplement: Dataset S1 — Open the file index.html to see a series of recordings made every hour between 13 Aug 2004 18:00 to 14 Aug 2004 06:00 Each file is presented as a wave file, with an audio player in index.html, and the spectrogram of the file. [file peerj-02-496-s001.zip › DatasetS1/index.html]

Series example


## Supplementary information - Series example

Site: El Yunque National Forest - Pico del Este. Coordinates: 18.27741, -65.76386

Every hour between 13 Aug 2004 18:00 to 14 Aug 2004 06:00

### **1800 hrs**

Audio file:  


Your browser does not support the audio element.

Spectrogram:

---

### **1900 hrs**

Audio file:   


Your browser does not support the audio element.

Spectrogram:

---

### **2000 hrs**

Audio file:   


Your browser does not support the audio element.

Spectrogram:

---

### **2100 hrs**

Audio file:   


Your browser does not support the audio element.

Spectrogram:

---

### **2200 hrs**

Audio file:   


Your browser does not support the audio element.

Spectrogram:

---

### **2300 hrs**

Audio file:   


Your browser does not support the audio element.

Spectrogram:

---

### **0000 hrs**

Audio file:   


Your browser does not support the audio element.

Spectrogram:

---

### **0100 hrs**

Audio file:   


Your browser does not support the audio element.

Spectrogram:

---

### **0200 hrs**

Audio file:   


Your browser does not support the audio element.

Spectrogram:

---

### **0300 hrs**

Audio file:   


Your browser does not support the audio element.

Spectrogram:

---

### **0400 hrs**

Audio file:   


Your browser does not support the audio element.

Spectrogram:

---

### **0500 hrs**

Audio file:   


Your browser does not support the audio element.

Spectrogram:

---

### **0600 hrs**

Audio file:   


Your browser does not support the audio element.

Spectrogram:

---

Spectrograms were generated up to 10kHz using a Hamming window and an FFT window size of 1024.
