# Supplementary figures and images for: Eleutherodactylus frogs show frequency but no temporal partitioning: implications for the acoustic niche hypothesis
Source: PeerJ. 2014 Jul 22;2:e496. doi: 10.7717/peerj.496 (PMC4121589; doi:10.7717/peerj.496)

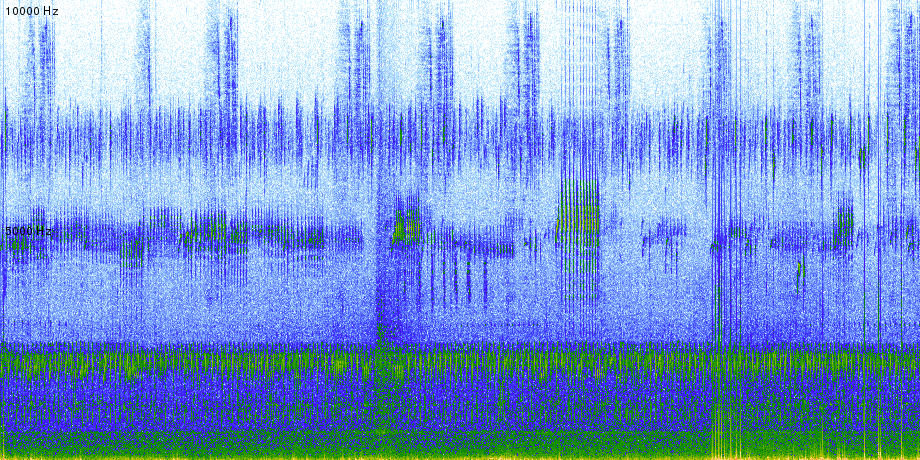

Supplement: Dataset S1 — Open the file index.html to see a series of recordings made every hour between 13 Aug 2004 18:00 to 14 Aug 2004 06:00 Each file is presented as a wave file, with an audio player in index.html, and the spectrogram of the file. [file peerj-02-496-s001.zip › DatasetS1/files/images/LINE_2004-08-13_18_01_31-large_s.png]

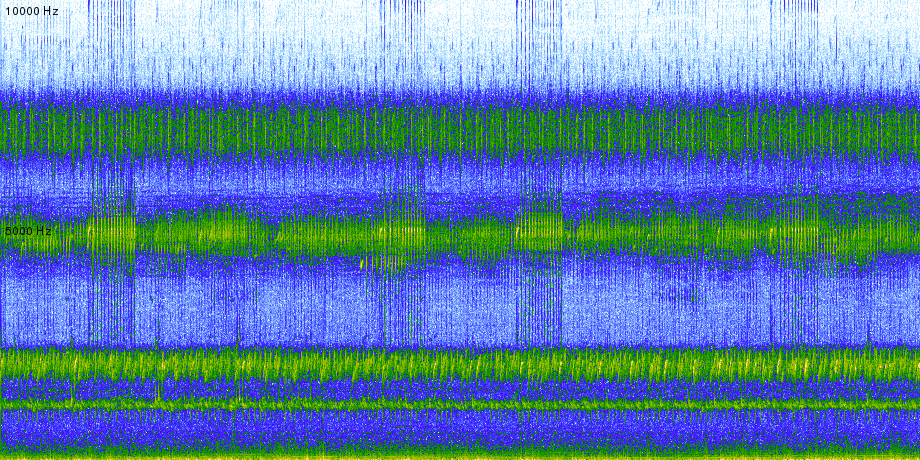

Supplement: Dataset S1 — Open the file index.html to see a series of recordings made every hour between 13 Aug 2004 18:00 to 14 Aug 2004 06:00 Each file is presented as a wave file, with an audio player in index.html, and the spectrogram of the file. [file peerj-02-496-s001.zip › DatasetS1/files/images/LINE_2004-08-13_19_01_30-large_s.png]

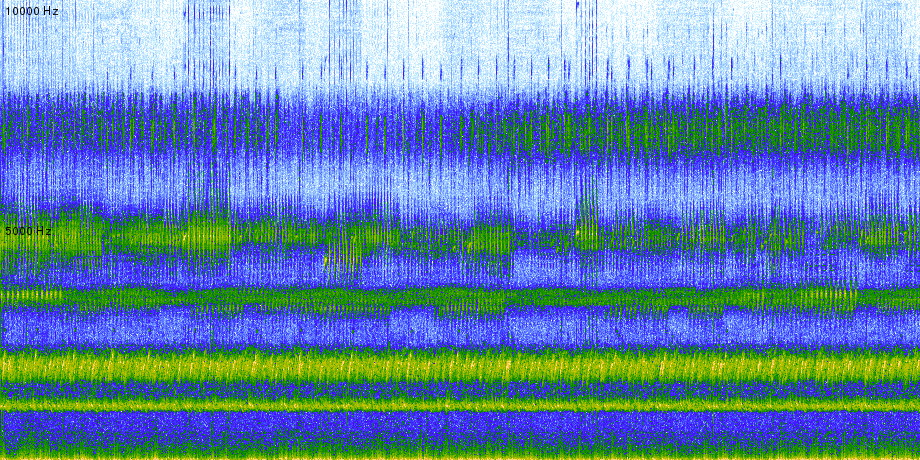

Supplement: Dataset S1 — Open the file index.html to see a series of recordings made every hour between 13 Aug 2004 18:00 to 14 Aug 2004 06:00 Each file is presented as a wave file, with an audio player in index.html, and the spectrogram of the file. [file peerj-02-496-s001.zip › DatasetS1/files/images/LINE_2004-08-13_20_01_29-large_s.png]

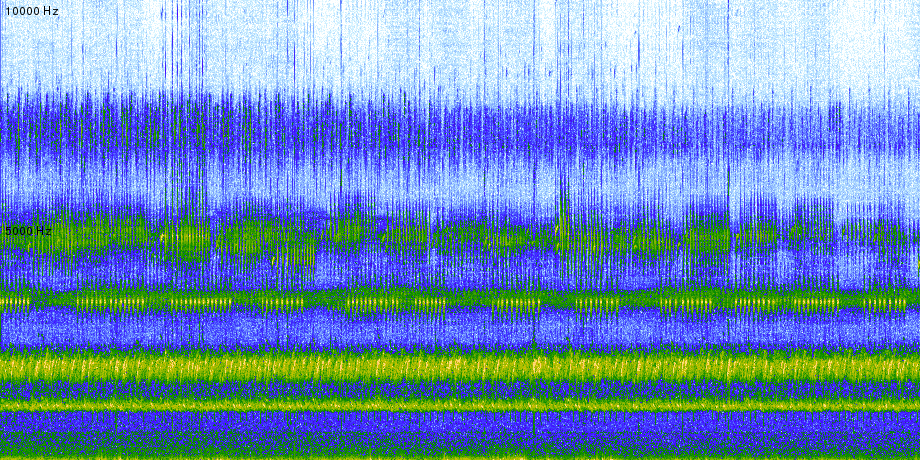

Supplement: Dataset S1 — Open the file index.html to see a series of recordings made every hour between 13 Aug 2004 18:00 to 14 Aug 2004 06:00 Each file is presented as a wave file, with an audio player in index.html, and the spectrogram of the file. [file peerj-02-496-s001.zip › DatasetS1/files/images/LINE_2004-08-13_21_01_29-large_s.png]

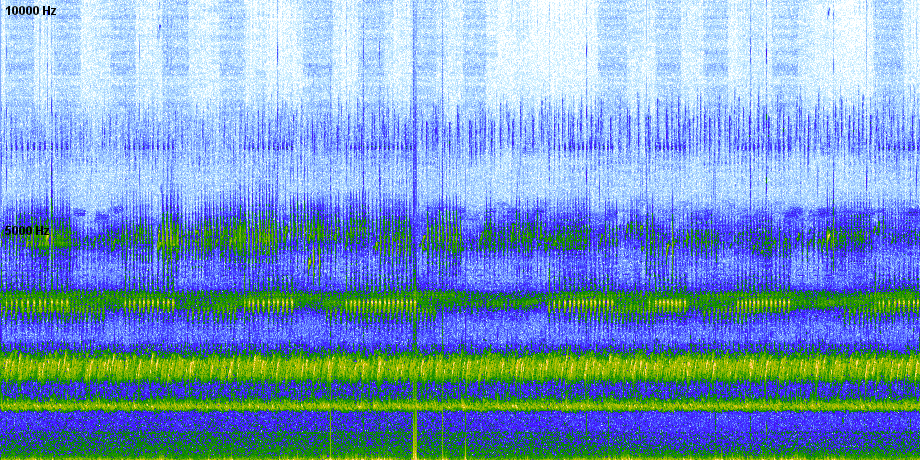

Supplement: Dataset S1 — Open the file index.html to see a series of recordings made every hour between 13 Aug 2004 18:00 to 14 Aug 2004 06:00 Each file is presented as a wave file, with an audio player in index.html, and the spectrogram of the file. [file peerj-02-496-s001.zip › DatasetS1/files/images/LINE_2004-08-13_22_01_28-large_s.png]

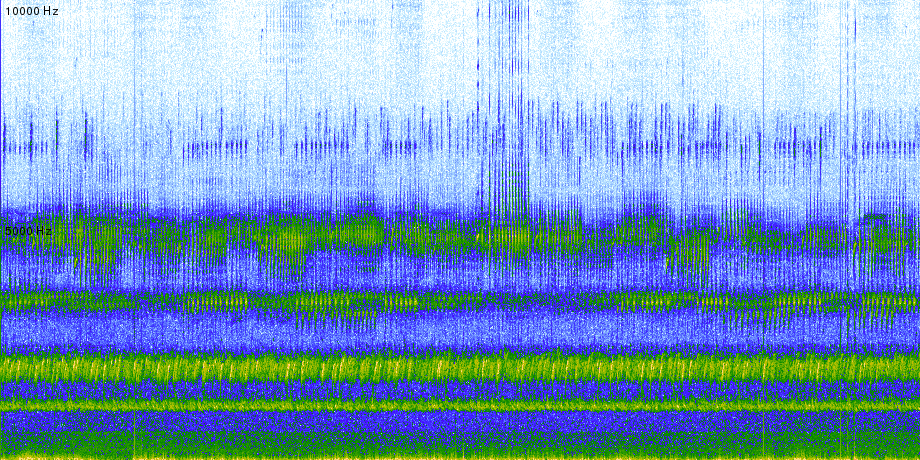

Supplement: Dataset S1 — Open the file index.html to see a series of recordings made every hour between 13 Aug 2004 18:00 to 14 Aug 2004 06:00 Each file is presented as a wave file, with an audio player in index.html, and the spectrogram of the file. [file peerj-02-496-s001.zip › DatasetS1/files/images/LINE_2004-08-13_23_01_28-large_s.png]

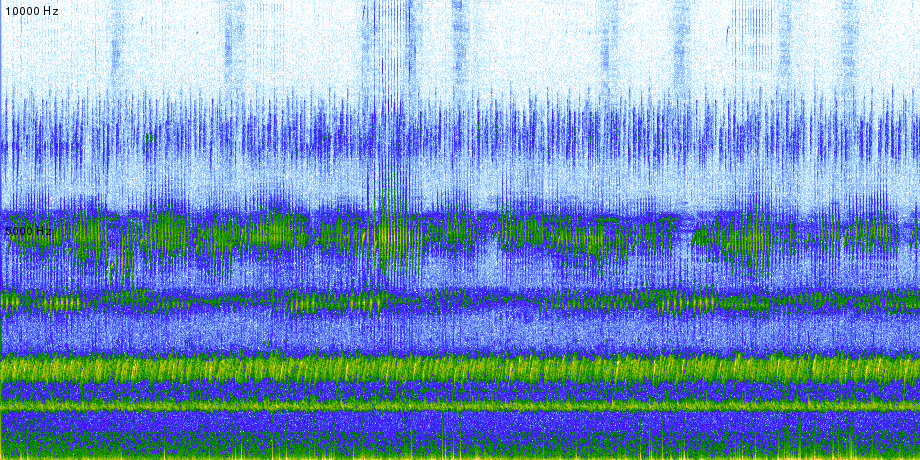

Supplement: Dataset S1 — Open the file index.html to see a series of recordings made every hour between 13 Aug 2004 18:00 to 14 Aug 2004 06:00 Each file is presented as a wave file, with an audio player in index.html, and the spectrogram of the file. [file peerj-02-496-s001.zip › DatasetS1/files/images/LINE_2004-08-14_00_01_27-large_s.png]

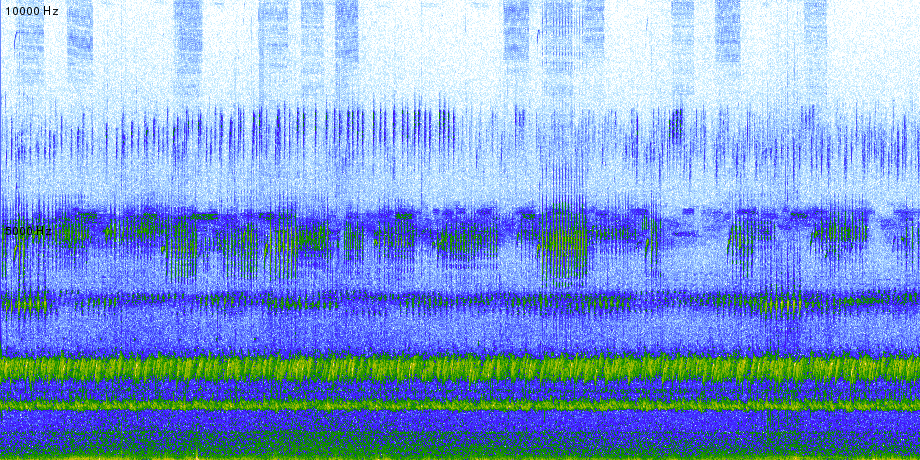

Supplement: Dataset S1 — Open the file index.html to see a series of recordings made every hour between 13 Aug 2004 18:00 to 14 Aug 2004 06:00 Each file is presented as a wave file, with an audio player in index.html, and the spectrogram of the file. [file peerj-02-496-s001.zip › DatasetS1/files/images/LINE_2004-08-14_01_01_27-large_s.png]

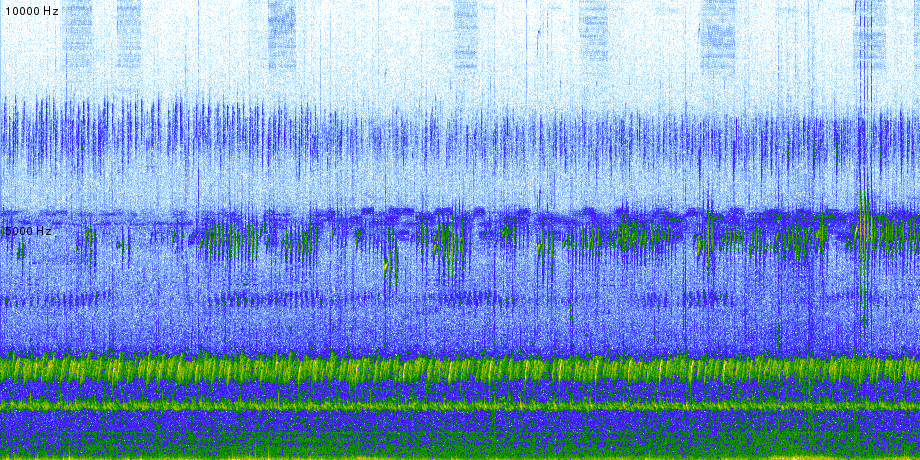

Supplement: Dataset S1 — Open the file index.html to see a series of recordings made every hour between 13 Aug 2004 18:00 to 14 Aug 2004 06:00 Each file is presented as a wave file, with an audio player in index.html, and the spectrogram of the file. [file peerj-02-496-s001.zip › DatasetS1/files/images/LINE_2004-08-14_02_01_26-large_s.png]

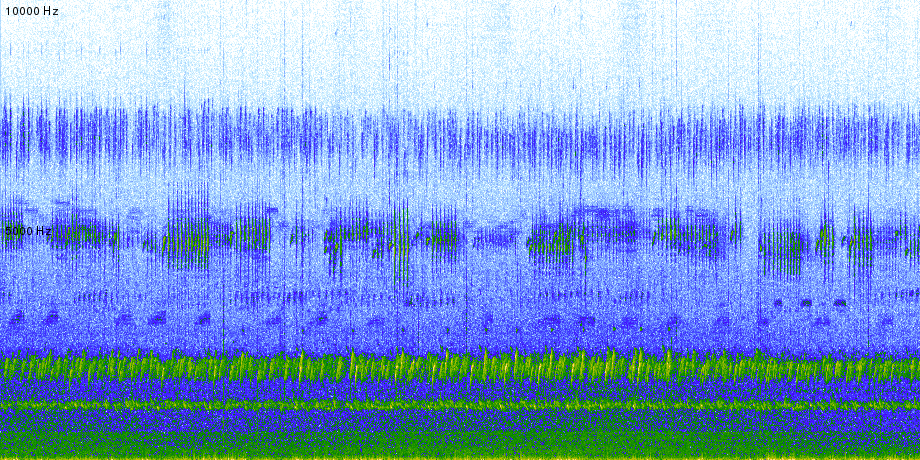

Supplement: Dataset S1 — Open the file index.html to see a series of recordings made every hour between 13 Aug 2004 18:00 to 14 Aug 2004 06:00 Each file is presented as a wave file, with an audio player in index.html, and the spectrogram of the file. [file peerj-02-496-s001.zip › DatasetS1/files/images/LINE_2004-08-14_03_01_25-large_s.png]

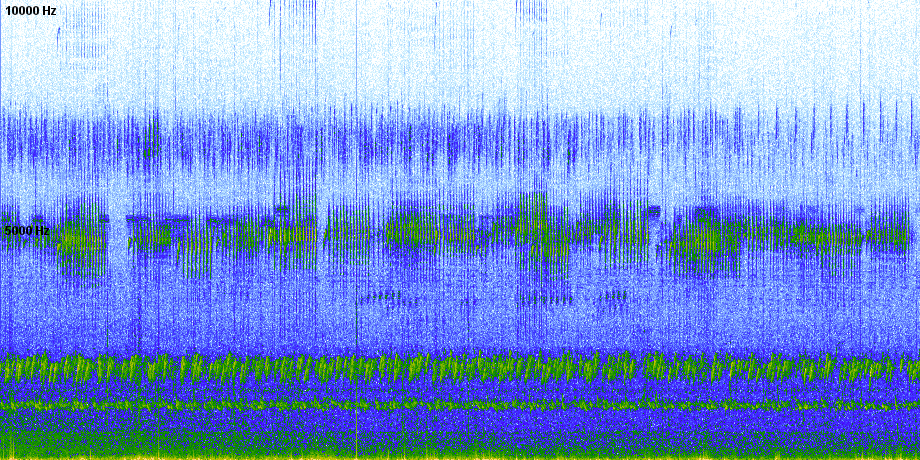

Supplement: Dataset S1 — Open the file index.html to see a series of recordings made every hour between 13 Aug 2004 18:00 to 14 Aug 2004 06:00 Each file is presented as a wave file, with an audio player in index.html, and the spectrogram of the file. [file peerj-02-496-s001.zip › DatasetS1/files/images/LINE_2004-08-14_04_01_24-large_s.png]

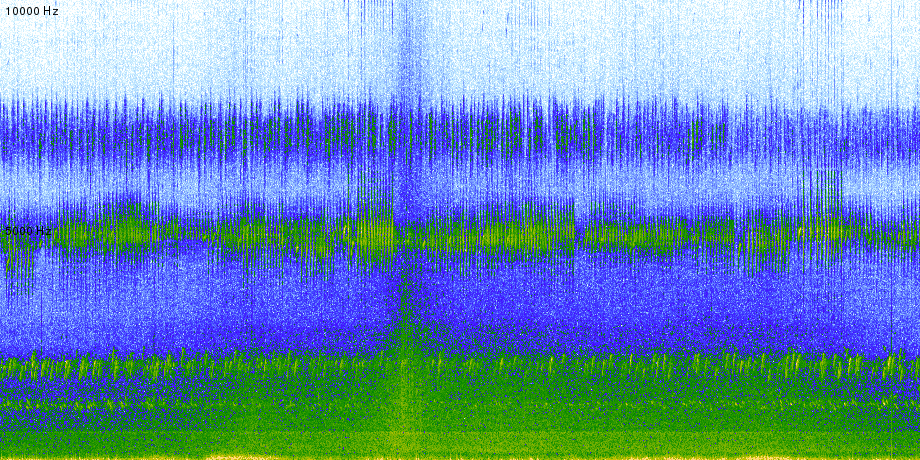

Supplement: Dataset S1 — Open the file index.html to see a series of recordings made every hour between 13 Aug 2004 18:00 to 14 Aug 2004 06:00 Each file is presented as a wave file, with an audio player in index.html, and the spectrogram of the file. [file peerj-02-496-s001.zip › DatasetS1/files/images/LINE_2004-08-14_05_01_24-large_s.png]

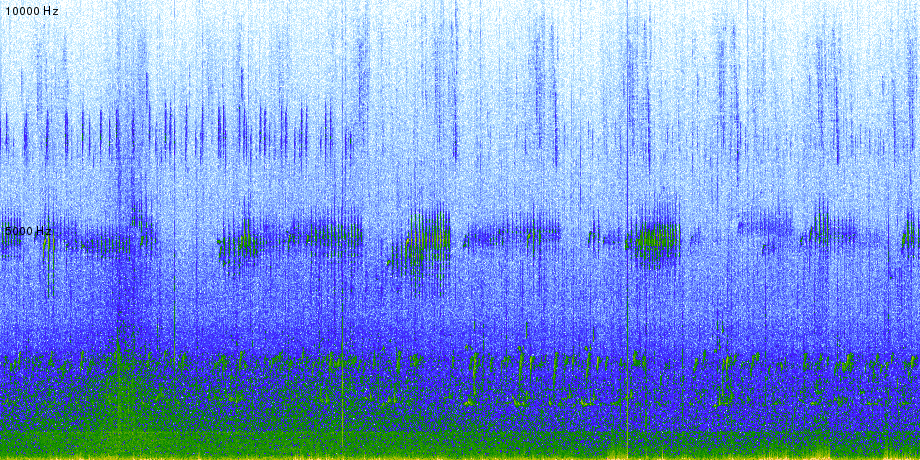

Supplement: Dataset S1 — Open the file index.html to see a series of recordings made every hour between 13 Aug 2004 18:00 to 14 Aug 2004 06:00 Each file is presented as a wave file, with an audio player in index.html, and the spectrogram of the file. [file peerj-02-496-s001.zip › DatasetS1/files/images/LINE_2004-08-14_06_01_24-large_s.png]
